# Pathological and bacteriological findings in sows, finisher pigs, and piglets, being culled for lameness

**DOI:** 10.1186/s40813-025-00463-4

**Published:** 2025-10-13

**Authors:** Magdalena Jacobson, Mika Berglund, Michelle Pettersson, Magdalena Sandström, Frida Matti, Marie Sjölund, Annette Backhans, Bjørnar Ytrehus, Stina Ekman

**Affiliations:** 1 Department of Clinical Sciences, SLU, Uppsala, Sweden; 2 Distriktsveterinärerna Rimbo, Rimbo, Sweden; 3 Distriktsveterinärerna Nerike, Lindesberg, Sweden; 4 Björkhyttan Stora Heleneberg 150, Lindesberg, Sweden; 5 Farm and Animal Health, Kungsängens Gård, Uppsala, Sweden; 6 Swedish Veterinary Agency, Uppsala, Sweden; 7 Norwegian Veterinary Institute, Ås, Norway; 8 Section for Pathology, Department of Biomedical Sciences and Veterinary Public Health, SLU, Uppsala, Sweden

**Keywords:** Arthritis, *Post mortem* examination, Antimicrobial susceptibility, Abrasions, Umbilical abscesses

## Abstract

**Background:**

Lameness is a common pig disorder. In the herd, a recurring, consistent aetiology is often suspected based on the age of the pig and herd-specific predisposing factors. The aetiology may be difficult to establish especially in larger animals, since the primary pathogenesis is obscured by delayed diagnosis, chronic changes and sequelae, and thorough investigation usually requires culling and transport to a pathological laboratory for necropsy. Thus, treatments are mostly based on a tentative diagnosis, commonly including antibiotics to eliminate infectious agents. The study aimed to investigate the aetiology of lameness in various age-categories of pigs, compare the tentative clinical diagnosis with post mortem findings, and discuss the findings in relation to the adequacy of antibiotic treatment.

**Results:**

In total, 29 sows, 45 finisher pigs, and 130 suckling piglets diagnosed with lameness were included. In all age categories, arthritis was the most common finding (13 sows, 30 finisher pigs, and 126 piglets, i.e. 45, 67, and 97% of the respective category). 86% (*n* = 25) of the sows and 71% (*n* = 32) of the finisher pigs had been treated with antibiotics. Of the *post mortal* lesions assessed to have an infectious aetiology, 99% were interpreted as chronic and eight sows, two finisher pigs and one suckling piglet had fractures. In six samples from finisher pigs, the most common finding was beta-haemolytic streptococci. In the suckling pigs, 223 samples from affected joints and 37 samples from macroscopically unaffected joints were subject to cultivation. The most commonly found bacterial species were *S. hyicus* (*n* = 73 isolates) and *S. dysgalactiae* ssp. *equisimilis* (*n* = 58). 46 of 64 *S. hyicus*-isolates tested were resistant to penicillin, whereas all 44 isolates of *S. dysgalactiae* ssp. *equisimilis* tested were sensitive to penicillin.

**Conclusion:**

In the majority of the cases, the findings at necropsy confirmed the clinical diagnosis. However, the rationale for antibiotic treatment must be questioned since 21 of 57 sows and finisher pigs (37%) treated with antibiotics were shown to suffer from lameness inflicted by non-infectious causes *post mortem*. Further, in all sows and finisher pigs, and in 83% (*n* = 104) of the suckling pigs with lesions being assessed to have an infectious aetiology at necropsy, the lesions were interpreted as being chronic. In piglets, *S. dysgalactiae* ssp. *equisimilis* or *S. hyicu*s were isolated in 64% of the samples. A majority of these isolates were susceptible to penicillin, still rendering this a valid drug for treatment in acute cases of arthritis.

## Introduction

Lameness, i.e. abnormal gait and/or inability to take full support on a leg, is a common disorder in pigs with reported prevalences ranging from 3.3 to 9.8% in suckling piglets, 1.9 to 19.7% in grower-finisher pigs, and from 4.5 to 38% in sows [1–6]. The differences in prevalence between herds and even countries seem to be related to a variation in predisposing factors such as; genetics, feed, flooring, or management routines [1, 4–8]. Further, the main causes of lameness seem to vary between different age-categories of animals. In addition, studies on lameness in larger animals are hampered by the difficulties of obtaining a proper aetiological diagnosis, since it usually requires culling and subsequent transportation of the carcass to a pathological laboratory, and thus implies extra costs, labour, and loss of the expected income from that animal [9].

In sows, several studies address the prevalence of and risk factors for lameness, but very few studies target the underlying pathological lesions. In a study on live animals, showing a prevalence of 9.7% lame sows, claw lesions were common and increased with age, whereas lameness decreased [10]. Studies on sows being euthanized or found dead demonstrated joint disease and arthritis to be the most prevalent *post-mortem* diagnosis, found in 9–36% of the sows [9, 11, 12]. Similarly, *post mortem* investigations of legs from sows culled due to lameness identified infectious arthritis and osteochondrosis as main causes [13, 14].

In the finisher pig, most studies focus on lesions found at the abattoir, the most common causes of “swollen joints” being inflammation due to osteochondrosis, and arthritis caused by infection with *Mesomycoplasma (Mycoplasma, M.) hyosynoviae* or *Erysipelothrix rhusiopathiae* [8, 15–17]. Few studies have addressed the overall causes of lameness during the rearing period in the finisher barn, but studies including bacteriological cultivation of synovial fluid aspirates indicate that *M. hyosynoviae* is found in only 20% of the cases and that a major proportion of the samples do not contain cultivable bacteria [15].

Studies on suckling piglets are scarce but mostly reveal a bacteriological aetiology, usually involving commonly occurring, opportunistic bacteria such as streptococci, staphylococci, *Trueperella (T.,* formerly *Arcanobacter* and *Actinomyces, A.) pyogenes,* and *Escherichia* (*E*.) *coli* [1, 18]. These bacteria, commonly found on the skin or in the environment, may invade the body through various injuries caused by e.g. surgical castrations, skin wounds or claw lesions caused by imperfect flooring, or umbilical infections in newborns [1, 19]. Further, bacteria carried in the tonsils may invade the vascular system, causing bacteraemia and subsequent localisation to various organs, including the joints [18, 20, 21].

Treatment of lameness in pigs includes antibiotics to eliminate infectious agents, and NSAIDs to reduce the inflammatory reaction, thereby alleviating pain [15, 22]. However, since a proper diagnosis is difficult to obtain in the live animal, treatment is mostly based on a tentative diagnosis and a best practices approach. Given the plethora of possible causes of lameness and the importance of prudent use of antibiotics, determining the causes and presumptive usefulness of antibiotic treatment of lame pigs should be prioritized.

Thus, this descriptive study aimed to investigate the aetiology of lameness in piglets, finishers and sows in Sweden, compare the tentative clinical diagnosis with *post mortem* findings, and discuss the findings in relation to the adequacy of antibiotic treatment.

## Material and methods

### Study design

The study was performed within the Swedish Veterinary Antibiotics Resistance Monitoring –farm animal pathogens (SVARMpat) programme from 2018 to 2021 and included three separate parts (I-III) targeting lame sows, finishers, and suckling pigs, respectively. The selection of farms and animals to be included in the study was made by ~ 20 veterinarians from the advisory company Farm and Animal Health and based on inclusion criteria specified by SVARMpat (see below). This company includes approximately 80% of the Swedish pig farms in their service. To ascertain that the lameness originated from the extremities, the animals underwent a clinical examination by the veterinarian. With the aid of the farmers or foremen, the veterinarians also filled in a short questionnaire to obtain some anamnestic information, albeit not intended for further analyses.

All animals were euthanized at the farm and following the clinical examination, the suspected diagnosis at the time of euthanasia was stated. In the sows and finisher pigs, the affected leg was identified and excised at the hip or scapula whereas in the suckling piglets, the entire carcasses were submitted. The specimens were marked individually and sealed in plastic bags at the farm, frozen within 24 hours, and stored at −20 °C prior to submission to the Section for Pathology at the Department of Biomedical Sciences and Veterinary Public Health, SLU, Uppsala, Sweden. At arrival, the specimens were stored at −20 °C and thawed the day before *post mortem* examination. Swabs for aerobic and anaerobic bacteriological cultivation were collected in all suckling pigs and in a few finisher pigs with a suspected joint infection. The gross lesions were photo-documented and the identity of the pig, the findings at necropsy, and the origin of the samples were recorded.

### Questionnaire

The questions were chosen according to each specific production form and included background information such as; the number of pigs on the farm and per unit, size and design of the pen and pen floor, litter material used, number of pigs per pen, outdoor access, and number of treatments and culling for lameness per batch or year. Further, the identity and age of the pigs, number of litters and reproductive status of the sow, breed, case history, duration of lameness, location of the lesion, other findings at the clinical examination, and any clinical suspicions on the cause of lameness classified as “fractures/external impingement”, “claw problems”, “infection/inflammation” and “others (what)” were stated. Further, information on treatments performed in the pig, were included.

I. Adult sows showing signs of lameness

During 2018, each veterinarian was to submit samples from a maximum of two breeding gilts or sows diagnosed with lameness. The sows were culled on the farm, and the leg presumed to be affected was excised and sealed in plastic bags at −20 °C before submission. Following thawing, each leg was thoroughly examined for swellings, wounds and lesions in soft tissues, bones and joints, and every joint was cut open. To determine the extent of the lesions, the joints were in most cases cleaved in the sagittal plane with a bandsaw.

II. Finisher pigs showing signs of lameness

During 2020, lame finisher pigs were targeted and each veterinarian was to submit samples from a maximum of two finisher pigs diagnosed with lameness per herd. Following thawing, the legs were visually inspected, and the joints were cut open. If osteomyelitis, osteochondrosis, or infection in the phalangeal joints was suspected, the soft tissue was removed, and the bone and joints were cleaved in the coronal or sagittal plane. Samples for bacteriologic cultivation were collected immediately after incision of the joints from five pigs with chronic arthritis or osteomyelitis.

III. Suckling piglets showing signs of lameness

In 2021, a maximum of three suckling piglets diagnosed with lameness per herd, and a maximum of eight piglets per veterinarian were identified, examined, and euthanized. The number of animals per herd was based on what could be expected to find at a specific herd visit. Runt pigs, pigs found dead, or pigs that had been treated with antibiotics, were excluded from the study. At the *post mortem* examination, the carcasses were visually inspected, and all lesions, contusions, swellings, and other signs of inflammation were noted. Based on the information provided in the questionnaire and on the results from the inspection, the two macroscopically most severely affected joints were selected for bacteriological examination. If only one joint was affected, the contralateral joint was also sampled. If no macroscopically affected joint could be identified despite the clinical diagnosis of lameness, all extremity joints above the distal phalangeal/digital joints were opened carefully to avoid contamination, and joints showing macroscopically visible signs of arthritis were selected. Following bacteriological sampling, the joint was inspected and specimens for histology were collected. Thereafter, all remaining extremity joints were similarly opened and inspected. Finally, the abdomen and thorax were opened, the body cavities and internal organs were inspected, and any deviations from normal were noted. Due to time constraints, meninges and inner ear were not inspected.

At the inspection, skin wounds were classified as “mild” (5–10 mm diameter, hair scraped off and/or superficial crusts in the dermis), “moderate” (20–40 mm diameter, covered by a thick crust in dermis), or “severe” (deep wound penetrating through dermis to underlying tissues). To assess the relationship between skin ulcers and arthritis in adjacent joints, the most severe ulcer adjacent to a joint was recorded. Claw lesions were classified as “mild” (sole abrasions or bleeding covering 10–30% of the sole, a small piece, i.e. less than ~5 mm diameter of the capsule torn off, a small wound in the coronary band, or a small bleeding in the accessory digits), “moderate” (sole abrasions or bleeding covering 30% or more of the sole, a larger piece, i.e. more than ~5 mm diameter of the capsule torn off, a large wound in the coronary band, or a larger bleeding in the accessory digits), or “severe” (a penetrating wound in the sole, capsule or accessory digits). Umbilical abscesses were categorised as “mild” (single abscesses < 3–5 mm diameter), “moderate” (single abscesses, approximately 6–10 mm in diameter), or “severe” (multiple abscesses, single abscesses approximately 15 × 5 mm in diameter, or abscesses involving urachus and umbilical vessels). The swelling of the joint was assessed as either “present or not present” in the first 50 piglets necropsied, but thereafter categorised as “mild”, “moderate” or “severe” if present. Cases of arthritis were grossly evaluated as acute (synovial response visible as hyperaemic, oedematous and velvet-like proliferation of the synovial membrane, increased amount of synovial fluid and/or pus in the joint cavity) or chronic (proliferation of the synovial membrane, thickened joint capsule, and/or erosions in the joint cartilage, with or without abscesses in close proximity to the capsule and joint). Joints with lesions suggesting both acute and chronic injuries were categorized as chronic-active arthritis.

### Microscopic examinations in suckling pigs

Slabs were sectioned transversally from the joint capsule, bone, and claw horn, fixed in 4% buffered formaldehyde, dehydrated, and routinely sectioned into histological specimens that were stained with hematoxylin and eosin (H&E). Sections from joints where *Staphylococcus (S.) hyicus* or *Streptococcus (S.) dysgalactiae* ssp. *equisimilis* had been identified in moderate or profuse growth in pure culture, were further studied regarding any association between lesions, identified bacteria, and the reported duration of the lameness. The results were compared to the findings in joints without any macroscopical lesions or bacterial growth. The inflammatory lesions were classified as fibrinous (presence of large amounts of fibrin in the synovial tissue and the joint cavity), purulent (cell infiltrates consisting of predominantly neutrophilic granulocytes in the synovial tissue and joint cavity), or fibrinopurulent (presence of large amounts of fibrin *and* cell infiltrates dominated by neutrophilic granulocytes). Further, the lesions were graded as acute (none or only a few macrophages, lymphocytes or plasma cells in the synovial tissue) or chronic (moderate to severe infiltration of macrophages, lymphocytes, plasma cells, and fibroblast activation in the synovial tissue and adjacent subsynovial connective tissue).

II-III. Bacteriological examinations

In the sows (study I), bacteriological sampling was not performed since most sows (*n* = 25) had previously been treated with antibiotics. In study II, samples for bacteriology were taken from untreated animals from joints where infection was suspected, and aerobic cultivation was performed at the Section for Bacteriology, Department of Biomedical Sciences and Veterinary Public Health, Faculty of Veterinary Medicine and Animal Husbandry, SLU, Uppsala, Sweden. In study III, aerobic and anaerobic cultivation and antimicrobial susceptibility-testing was performed at the reference laboratory, the Swedish Veterinary Agency (SVA) in Uppsala. The joint was incised by a sterile scalpel blade and a swab (Amies W/CH, Copan, Brescia, Italy) was inserted through the incision into the joint and rubbed against the synovial membrane. In the first 60 suckling pigs, the selected joints were seared with a Bunsen burner for 2–4 seconds before being cut open with a sterile scalpel blade. Based on the results from the cultivation which indicated contamination in some cases, the procedure was improved for the remaining 70 pigs. Here, the skin above the joint was disinfected with 70% alcohol before flame disinfection and incision as described above. Two swabs per joint were collected and the contralateral joints were sampled before affected joints. Each swab was marked with the date, individual, leg, and joint, packed in plastic and transferred to the laboratory on the same day.

The swabs for aerobic cultivation were analysed according to accredited standard methods (ISO 17,025) used at SVA. Briefly, the swabs were streaked on 5% horse blood agar, lactose purple agar, and hematin agar with yeast extract (SVA, Uppsala, Sweden), incubated aerobically at 37 ± 1 °C and examined for bacterial growth after 24 and 48 hours. If no growth occurred, the sample was enriched in serum broth overnight, and thereafter incubated on bovine blood agar (SVA) for an additional 48 hours at 37 °C. In case of bacterial overgrowth, colistin-sulphate oxolinic acid blood agar was used as well. *E. coli* was identified by the ability to ferment lactose and by the production of indol. *Streptococcus* ssp. were identified by Matrix-Assisted Laser Desorption/Ionization Time of Flight Mass Spectrometry (MALDI-ToF; Bruker Daltonik Gmbh, Bremen, Germany) [23] and *S. canis*, *S. pyogenes* and *S. dysgalactiae* ssp*. equisimilis* were identified by Lancefield grouping (Streptex^TM^ Rapid Latex Agglutination Test, ThermoFisher Scientific, Waltham, MA, USA). Other bacteria were identified to the species level by MALDI-ToF. Antimicrobial susceptibility was tested by broth microdilution in routine diagnostics at SVA following recommendations in ISO 20,776–1:2019, and minimum inhibitory concentrations (MIC) as the lowest concentration where visible growth was inhibited, were determined. Three different custom-made panels were used for *Staphylococcus* ssp. and *Streptococcus* ssp., *E. coli* and *Actinobacillus* ssp. isolates. Isolates of *Staphylococcus* ssp. were also tested for the production of beta-lactamase using the Cefinase test (BD BBL^™^ Cefinase^™^ paper discs, Becton Dickinson, Franklin Lakes, NJ, USA). Isolates were regarded as resistant or susceptible based on clinical breakpoints issued by the Clinical and Laboratory Standards institute VET01S (CLSI VET01S, 7th edition, Performance standards for antimicrobial disk and dilution susceptibility tests for bacteria isolated from animals) or CLSI M100 (CLSI M100, 34th edition, Performance standards for antimicrobial susceptibility testing). When no CLSI breakpoints were available, epidemiological cut-off values by EUCAST (European committee on antimicrobial susceptibility testing; https://mic.eucast.org) were used. Staphylococci were determined as resistant to penicillin by a positive cefinase test.

The swabs for anaerobic cultivation were streaked on two Fastidious Anaerobic Agar plates (FAA, SVA) within 24 hours. One plate was incubated anaerobically in jars using Thermo Scientific™ Oxoid AnaeroGen 2.5 L Sachets (OXOID, Basingstoke, Great Britain) and an Anair indicator (bioMérieux, Marcy-l’Etoile, France) at 37 °C for 48 hours. The second plate was incubated aerobically to investigate the presence of facultative anaerobic bacteria. The isolates were typed by MALDI-ToF. Antimicrobial susceptibility testing was not performed on these isolates.

## Results

All farms included in this study practised”conditional medical treatment”, a practise that is regulated by law and implies that animal caretakers, following a regulated course-work and under the supervision of the herd veterinarian, are allowed to induce specified treatments upon specified clinical signs (The Swedish Board of Agriculture, No 2023:21, D 35/L41, http://www.jordbruksverket.se).

I. Adult sows showing signs of lameness

In total, 32 legs from 29 sows fulfilled the inclusion criteria and were subject to necropsy, including 15 forelimbs and 17 hindlimbs. In two cases, no macroscopic lesions were found.

In two cases, the duration of lameness was not stated. Six out of 29 sows (21%) had been lame for less than one week before being culled, 18 sows (62%) had been lame for 2–4 weeks, and three sows (10%) had been lame for more than six weeks. Ten sows (34%), clinically suspected with fracture at the day of euthanasia, had reportedly showed signs of lameness for one week to one month before being euthanized, and seven of these sows had also been treated with antibiotics.

The group with the highest number of sows (*n* = 14, 48%) were first-parity sows that had been culled after weaning, 42% (*n* = 12) of the sows were of parity 2 to 4, and three sows (10%) have had more than five litters. Lameness was not observed at any specific location or period in the reproductive cycle. For underlying aetiologies, however, sows diagnosed with fracture (*n* = 4, 50%) were overrepresented in the breeding unit. Data on the herd prevalences of lameness in sows were not available.

The main macroscopic lesions judged as the cause of the lameness in 27 sows are summarised in Table 1.

**Table 1:** Main macroscopic lesions in joints from lame sows

Main lesion	Number of animals (%)	Macroscopic findings at *post mortem* examination. (n) = Number.
Fracture	8 (30)	Acute (n=2) and chronic fracture in femur with chronic osteoarthritis in the hip joints (n=2). Chronic intraarticular fractures in humerus with secondary osteoarthritis in the shoulder joints (n=3). Chronic fracture of humerus (n=1).
Abscess and tendinitis	1 (4)	Left hindleg at the level of the dew claws
Chronic arthritis with osteomyelitis	6 (22)	Chronic arthritis of the pastern or coffin joints with osteomyelitis and sequester formation (n=4). Chronic arthritis with osteomyelitis of the hock joint (n=1) and intraarticular fibrous fusion of the shoulder joint (n=1).
Chronic arthritis with osteomyelitis and abscesses	3 (11)	Chronic fibrinopurulent to purulent arthritis of the pastern and coffin joints with osteomyelitis, abscess formation and ulceration (n=2). Chronic purulent arthritis of the shoulder joint with osteomyelitis in proximal humerus (n=1).
Chronic arthritis with abscesses and tendinitis	4 (15)	Chronic purulent arthritis with tenosynovitis and abscess formation in the fetlock joint (n=1), coffin joint (n=1), carpal joint (n=1), and hip joint (n=1).
Chronic capsulitis and synovitis	1 (4)	Chronic fibrinous haemorrhagic capsulitis and synovitis of the stifle joint
Granulomatous desmitis and periostitis	1 (4)	Granulomatous desmitis and periostitis of the proximal plantar metacarpus III
Osteochondrosis/osteoarthritis	3 (11)	Osteochondrosis and osteoarthritis in the medial femur condyle of the hip joint (n=2) and in the medial humerus condyle of the elbow joint (n=1).

The main macroscopic lesions found at post mortem investigation of legs from 27 lame sows euthanized at the farm. The affected legs were excised at the hip or scapula, frozen at −20 °C, and submitted for necropsy

**Fig. 1:**
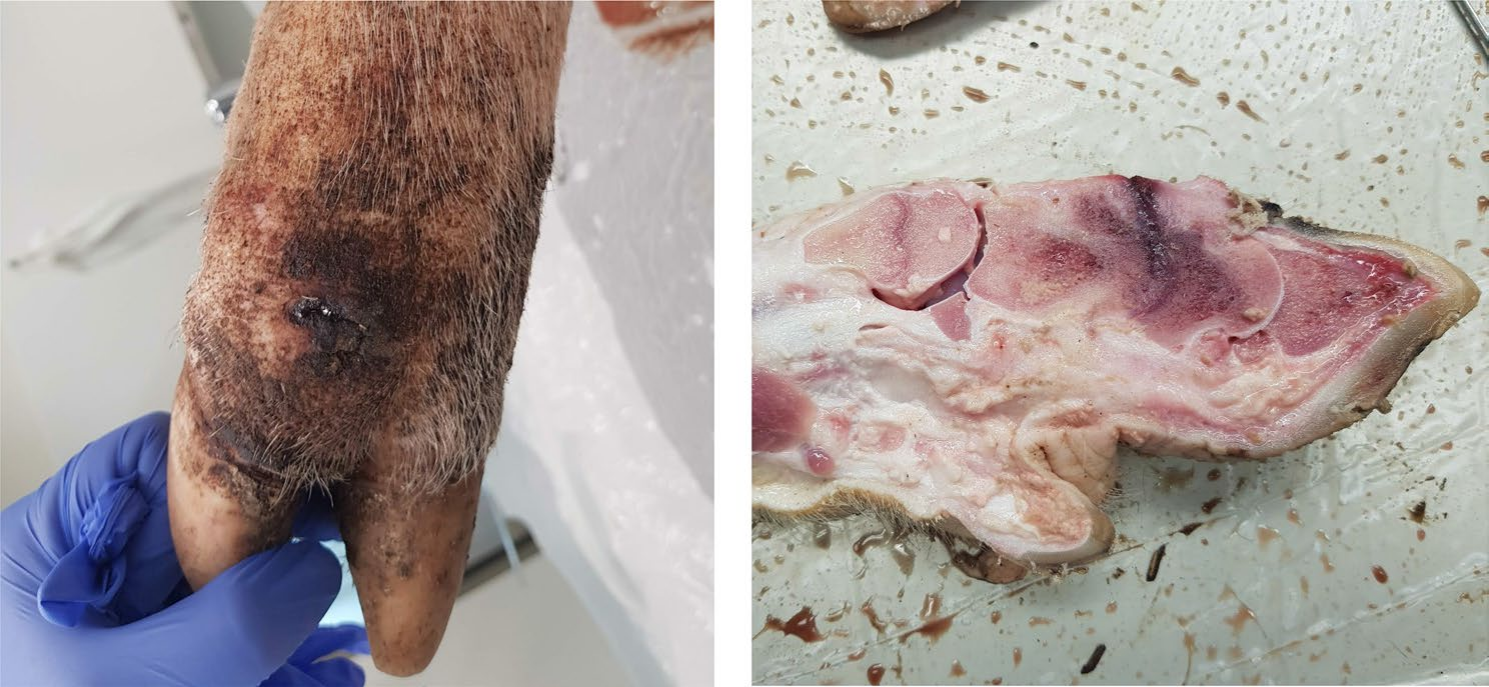
Lesions found at necropsy in a sow suffering from lameness. Findings at necropsy in a sow suffering from chronic arthritis in the lateral proximal interphalangeal joint, osteomyelitis and sequester in the intermediate and distal phalanges, deformed joint cavity with complete destruction of the proximal and distal cartilage, haemorrhages and granulation tissue formation. A fistulae communicating with the joint has developed (photo: Michelle Pettersson)

At necropsy, fractures was found to be the cause of lameness in eight cases, assessed as acute with dissecting haemorrhages, or chronic, based on the presence of cartilage- and bone callus. In fifteen sows (52%), an infectious aetiology was suspected. Thirteen sows (45%) suffered from chronic arthritis and in three of these, abscesses with fistulae communicating with the joint, were also found (Fig. 1). In six cases (21%), osteochondrosis dissecans and osteoarthritis of the elbow and stifle joints were also found but were not judged as the primary cause of lameness.

In ten cases (34%), a clinical suspicion of fracture was at hand, and the tentative diagnosis was correct in five of these, whereas one sow (3%) suffered from osteochondrosis dissecans, 2 (7%) from chronic purulent arthritis and osteomyelitis, 1 (3%) from osteoarthritis, and one (3%) from chronic synovitis and capsulitis. Claw lesions were suspected clinically and confirmed by pathological examination in eight (28%) cases. Infectious arthritis was suspected clinically in eight (28%) cases, four of these were correctly diagnosed, whereas two were in fact fractures, one turned out to be granulomatous desmitis and periostitis, and one case was without detectable lesions on *post mortem* examination. In three cases (10%) where osteochondrosis was suspected, one case was confirmed as the cause of lameness, while one was judged to be caused by chronic purulent arthritis and osteomyelitis, and one case was without post mortal findings. Among the eight sows diagnosed with fractures at necropsy, five cases had been correctly diagnosed clinically by the veterinarians, whereas arthritis were suspected in three cases.

The majority of the cases (*n* = 25; 86%) had been treated with antibiotics, solely or in combination with NSAIDs, given intramuscularly. In 13 cases (48%), a non-infectious aetiology was found at necropsy. The number of treatment days varied between three and 13 (mean eight days, median nine days). In 24 of the cases treated with antibiotics (96%), procaine-benzylpenicillin was the drug of choice and in one case, tetracycline had been used. One case had also been treated with selenium. One sow, where no specific lesions were found at necropsy, had been treated with NSAIDs only. In one sow with fracture, no treatment had been initiated.

II. Finisher pigs showing signs of lameness

In total, 56 legs from 45 finisher pigs were submitted for examination, including 33 forelimbs and 23 hindlimbs. In six legs from five individuals, no macroscopic lesions were identified, and these were excluded from further analyses. Thus, 40 pigs were included in the analyses.

**Fig. 2:**
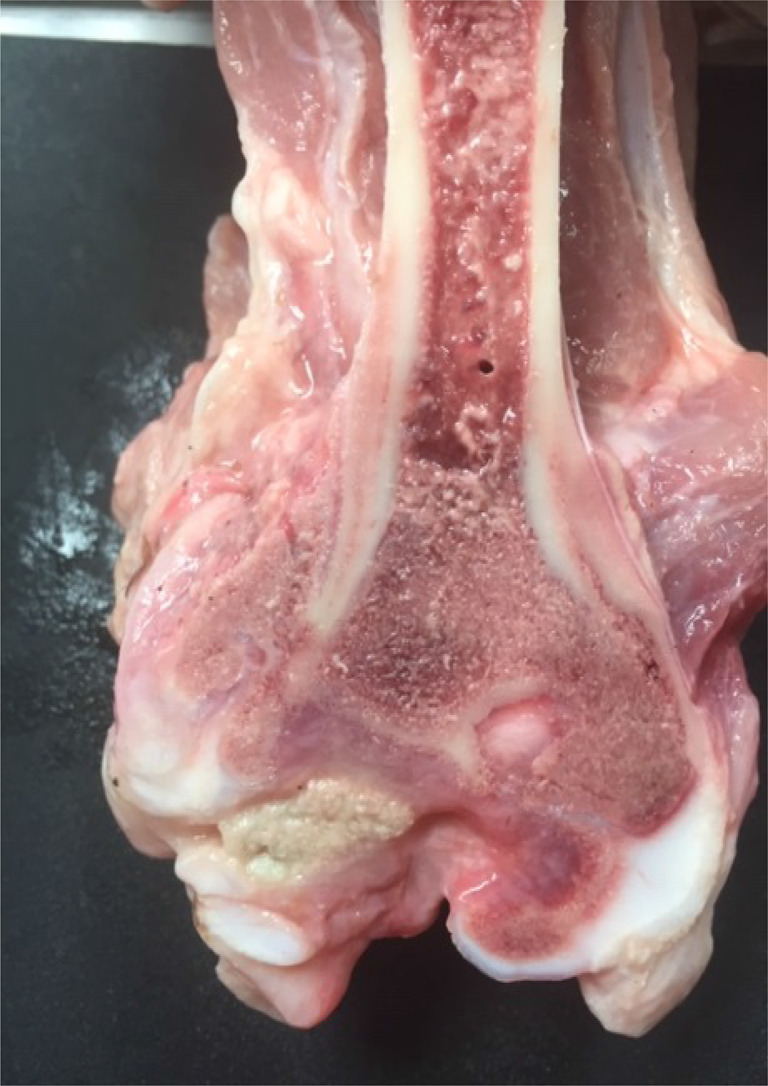
Lesions found at necropsy in a finisher pig suffering from lameness. Findings at necropsy in a finisher pig, showing chronic purulent arthritis in the elbow joint and osteomyelitis in the lateral humeral epiphysis (photo: Johanna Gripsborn)

Among the 45 cases submitted for examination, the clinical diagnosis was confirmed by *post mortem* examination in 27 cases (60%). The most common clinical diagnosis was “infection” as stated in 26 cases and being confirmed at necropsy in 20 cases (77%). In eleven cases with a clinical suspicion of fracture, 9 (82%) were diagnosed as chronic purulent arthritis or chronic purulent arthritis with osteomyelitis *post mortem* (Fig. 2). “Claw problems” was clinically diagnosed in 4 cases, being confirmed in 3 cases (75%) *post mortem*, the remaining cases being categorised as “other” in the clinical diagnosis.

The pigs had been in the finisher unit from between five days to 22 weeks prior to euthanasia (mean seven weeks), corresponding to an approximate mean age of 17 weeks. The reported prevalence of lameness in the herds varied between 1 and 16% per batch, as estimated from the last two batches in each farm. In 12 cases (27%), the duration of lameness was not stated. In three cases (7%), the pig was euthanized on the day of detection. In the remaining 30 cases (66%), the lameness had lasted from two days to eight weeks (mean nine days, median seven days).

The macroscopic lesions are summarised in Table 2.

**Table 2:** Main macroscopic lesions in joints from finisher pigs

Lesion	Number of animals (%)	Macroscopic findings at *post mortem* examination (*n* = number)
Fracture	2 (5)	Acute fracture in tibia and fibula (*n* = 1). Chronic fracture in the elbow joint with necrotic bone tissue (*n* = 1).
Osteomyelitis with abscesses	1 (2)	Purulent osteomyelitis in the medial phalanx and abscess formation in the front limb.
Purulent arthritis	9 (23)	Chronic purulent arthritis in the stifle joint (*n* = 3), the tibio-tarsal joint (*n* = 1), the shoulder joint (*n* = 1), the elbow joint (*n* = 4) with ulceration in the joint cartilage (*n* = 2), six cases also including abscesses in joint capsule and adjacent tissue.
Purulent arthritis with osteomyelitis	12 (30)	Chronic purulent arthritis and osteomyelitis in the hip joint (*n* = 1), the shoulder joint (*n* = 1), the stifle joint (*n* = 2), the elbow joint (*n* = 4), the metacarpal-phalangeal joint (*n* = 1), the radio-carpal joint (*n* = 1), the shoulder and elbow joint (*n* = 1), and the shoulder and tibio-tarsal joint (*n* = 1). Ten cases also included abscesses in joint capsules and adjacent tissues. In one case the entire caput femori was destructed.
Chronic arthritis or osteoarthritis	9 (23)	Chronic proliferative arthritis in the stifle joint (*n* = 2), the elbow joint (*n* = 3), the hip joint (*n* = 1), and the radio-carpal joint (*n* = 1), chronic osteoarthritis in the elbow joint (*n* = 1), and chronic arthritis in the elbow joint (*n* = 1), four cases also included abscesses in joint capsules and adjacent tissues, and ankylosis in two cases.
Claw lesions	5 (12)	Long, deformed claws, with dark, loose horn and healed abscesses (*n* = 3), unevenly sized claws and superficial horn lesions (*n* = 1), fibrotic scar of the coronary band (*n* = 1).
Physeal osteochondrosis	2 (5)	Osteochondrosis with microfractures in the metaphyseal growth plate in ulna.

The main macroscopic lesions found at post mortem investigation of legs from 40 lame finisher pigs euthanized at the farm. The affected legs were excised at the hip or scapula, frozen at −20 °C, and submitted for necropsy

32 of the 45 pigs (71%) had been treated with antibiotics for up to ten days (mean five days). In eight of these (25%), a non-infectious cause was at hand at necropsy. Three pigs (7%) had been treated with NSAIDs only, and in ten cases (22%), no treatment had been given.

III. Suckling piglets showing signs of lameness

In total, 130 pigs were submitted. In four of 129 pigs that presented with “swollen joints” at inspection, arthritis could not be confirmed, thus, 126 pigs were included in further analyses. In addition, information on the clinical suspicion was missing in eight cases. An infectious aetiology was clinically suspected in 110 pigs (90%). Eleven pigs (9%) were judged to suffer from “claw problems”, all being judged as infectious claw lesions at necropsy. In six cases, a clinical suspicion of fracture and/or infection was at hand. In one case, the lameness was clinically deemed to be caused by “wounds”. As stated in the inclusion criteria, none of the pigs had been treated with antibiotics.

Data on the herd prevalences of lameness in suckling pigs were not available. 45 pigs (35%) had developed lameness during their first week of life, 49 (38%) were one to two weeks old, 23 (18%) two to three weeks old, and 10 (8%) three to four weeks old at submission. In three pigs, information about age was missing.

According to the anamnestic information, 61 pigs (47%) had showed signs of lameness for less than one day, 40 pigs (31%) had been lame for one day, and 17 pigs (13%) for more than one day. In twelve cases (9%), this information was not provided.

The macroscopic findings in the most severely affected joint of each pig at necropsy are summarised in Table 3.

**Table 3:** The main macroscopic lesions found at *post mortem* investigation of 126 lame piglets

Lesion*	Number of animals (%)	Macroscopic findings at *post mortem* examination (*n* = number)
Fracture	1 (1)	Chronic fracture of metatarsal bone and arthritis in other joints.
Acute purulent arthritis	2 (2)	Synovial response with hyperaemia and purulent exudate
Chronic-active arthritis	75 (60)	Synovial response with purulent exudate, synovial proliferation, fibrosis of joint capsule, erosions in joint cartilage and abscesses.
Chronic arthritis	29 (23)	Fibrosis of joint capsule, erosions in joint cartilage and abscesses within or adjacent to the joint capsule without purulent exudate, increased joint fluid or synovial response.
Claw lesions	126 (100)	Moderate lesions in 62, severe lesions in 16. Of 37 piglets with arthritis in phalangeal joints, 32 also had lesions in the adjacent claw tissues.
Skin wound	114 (90)	Most common around carpal joints, metacarpal joints and lateral to the tibiotalar joint.

The piglets were euthanized at the farm, the carcasses were frozen at −20 °C and submitted for necropsy. The thawed carcasses were examined for joint lesions in all limbs and the internal organs were inspected

*A pig could suffer from several of the lesions described above, and thus be included in more than one group

**Fig. 3:**
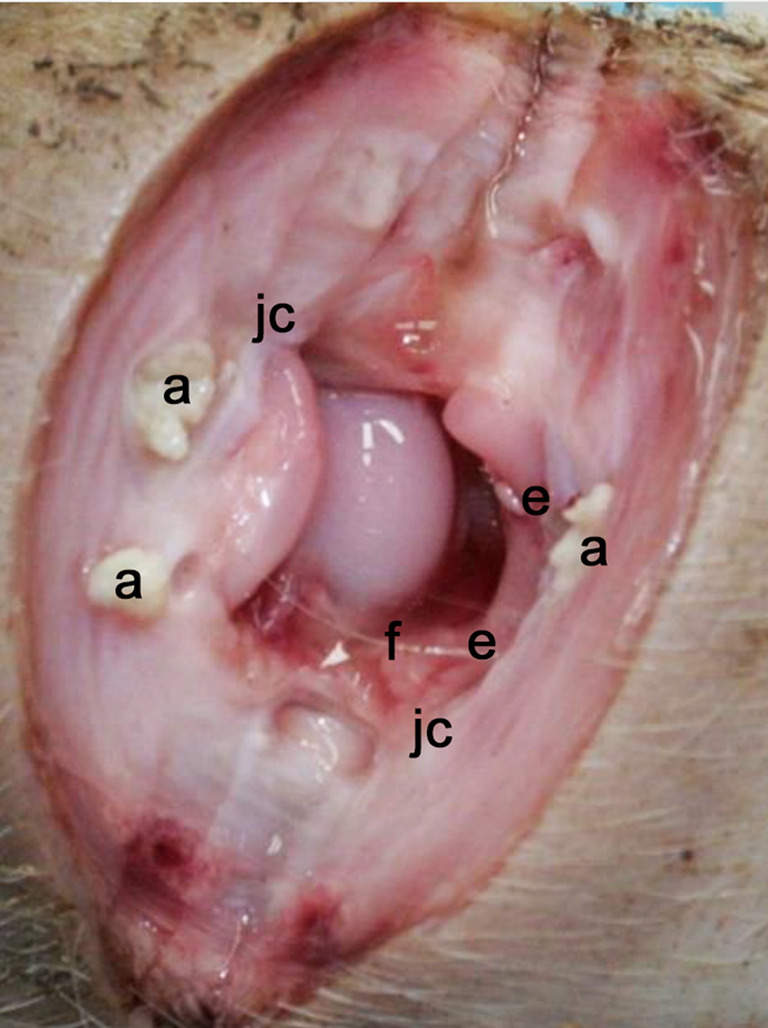
Lesions found at necropsy in a suckling pig suffering from lameness. Dorsal view of an opened hock (tibiotalar) joint of a suckling pig, showing lesions consistent with chronic arthritis, with erosions in the joint cartilage and exposed subchondral bone (e), moderate fibrinous exudate in joint cavity (f), mild periarthritis, and thickened joint capsule (jc) with abscesses in the capsule (a). Bacterial examination showed moderate growth of streptococcus dysgalactiae ssp. equisimilis in sparse mixed flora. According to the anamnestic information, the pig had been lame for one day (photo: Mika Berglund and Magdalena Sandström)

At necropsy, 125 pigs were shown to suffer from arthritis (96%). In 116 pigs (89%), arthritis was diagnosed in more than one joint and in 70 pigs (54%), five or more joints were affected. The most common location of arthritis was the carpal joint (*n* = 134, 51.5% of 260 carpal joints investigated) and the hock joint (*n* = 126, 48.5% of 260 hock joints investigated; Fig. 3).

One pig had an old fracture of the metatarsal bone, in addition to arthritis in other joints. Two pigs had only acute lesions, macroscopically characterised by synovial proliferation and an increased amount of purulent synovial fluid. Acute lesions with chronic elements, characterised by synovial proliferation, increased amount of purulent synovial fluid, thickened joint capsule, erosions in the joint cartilage, and abscesses in adjacent tissue, were observed in 75 pigs (60%; Fig. 3).

Chronic lesions characterised by thickened joint capsules, erosions in the joint cartilage, and abscesses in adjacent tissue, were found in 29 pigs (23%). Almost all pigs (114 of 130; 87.7%) had superficial skin wounds but these were most often not found in direct association with infected joints, however, in 95 pigs (73.1%), skin abrasions were also located close to joints with arthritis, most commonly dorso-distally to the carpal or fetlock joints, or caudo-laterally-distally to the hock joint. In 31 (33%) of these, the wounds were penetrating through the dermis. Claw lesions were noted in 126 pigs, the majority (*n* = 98; 78%) graded as “moderate” or “severe”. In total, 37 pigs (29%) had arthritis in the distal phalangeal joints, and 32 of these also had lesions in the claw, 16 of which were graded as “severe”. Umbilical abscesses were found in 68 pigs (52.3%). Twelve pigs with such abscesses deemed as “moderate” or “severe”, had arthritis in three or more joints. In 53 pigs (42%), inflammation was also found in other parts of the body, such as abscesses along the spermatic cord, enlarged lymph nodes, and foci of fibrinous exudate in the peritoneal or pleural cavity. All of these 53 pigs had cutaneous lesions on the legs, and 29 of them also had umbilical abscesses.

### Microscopic examinations

From the 130 suckling pigs, 22 sections from the joint capsule were included in the examinations, whereas seven sections were excluded because of the inaccurate depth of the specimen. In 11 of these cases, *S. dysgalactiae* ssp. *equisimilis* had been isolated, and in eight cases, *S. hyicus* had been isolated. The findings were compared to three sections from the capsule of macroscopically unaffected joints without any bacteriological findings after cultivation, displaying a thin layer of synoviocytes facing the joint cavity, and without any inflammatory cell infiltrates. One of the two pigs macroscopically assessed as suffering from chronic arthritis, was shown to suffer from acute arthritis by histology. Joints infected with *S. hyicus* were affected by fibrinopurulent acute to chronic arthritis and in one case, fibrinous chronic arthritis. Joints from which *S. dysgalactiae* ssp. *equisimilis* were isolated, displayed chronic, fibrinopurulent or chronic, fibrinopurulent, proliferative arthritis, and in two cases, also abscesses in the joint capsule.

II-III. Bacteriologic examinations

In the finisher pigs, bacteriologic cultivation from three chronic purulent, and one non-purulent arthritis, demonstrated growth of beta-haemolytic streptococci (*S. canis*/*S. dysgalactiae* spp). One case of chronic osteomyelitis with sequestration showed *S. aureus* in pure culture, and one case of purulent osteomyelitis demonstrated *T. pyogenes*. In one pig, bacteriologic cultivation from a chronic purulent lesion was negative.

In the suckling pigs, samples were collected from 260 joints, whereof 223 (86%) were macroscopically judged as arthritis and 37 were macroscopically unaffected joints. Four samples from two pigs were mislabelled and thus excluded from the analyses. Of the remaining 256 samples, aerobic bacteria were cultured in 204 joints (80%) from 102 pigs, whereas 52 joints (20%) were negative by cultivation. Bacterial growth was demonstrated in 23 (62%) of the macroscopically unaffected joints, however, a mixed bacterial flora was a common finding (50%), particularly in samples from the fetlock and phalangeal joints. In the samples from joints where the skin had been disinfected with 70% alcohol and burnt before being incised, approximately 29% (*n* = 40) of the samples demonstrated a mixed bacterial growth by cultivation, and in the samples not being disinfected before burning, 64% (*n* = 75) demonstrated a mixed bacterial flora. The most commonly found pathogenic species were *S. hyicus* (*n* = 73 isolates) and *S. dysgalactiae* ssp. *equisimilis* (*n* = 58). *S. aureus* was detected in 11 joints. Other pathogenic isolates included beta-hemolytic S*treptococcus* spp., *S. suis*, *E. coli* and *Actinobacillus* sp. and the findings of known pathogenic isolates are summarised in Table 4.

**Table 4:** Known pathogenic bacterial species* present in joints from lame piglets

Bacterial species	Isolated from joints with macroscopic changes	Isolated from macroscopically unaffected joints	Total
*S. dysgalactiae* ssp. *equisimilis*	56	2	58
*S. hyicus*	65	8	73
*S. aureus*	11	0	11
Beta-hemolytic streptococci	17	1	18
*S. suis*	5	1	6
*E. coli*	11	1	12
*Actinobacillus* sp.	3	1	4

Clinically relevant bacterial species present in joints from pigs suffering from lameness as identified by aerobic cultivation of samples obtained at necropsy

*Zoric, M., Nilsson, E., Mattsson, S., Lundeheim, N., Wallgren, P. 2008. Abrasions and lameness in piglets born in different farrowing systems with different types of floor. Acta Vet. Scand.50:37, 1–9. Doi: 10.1186/1751–0147–50–37 and Hill, B. D., Corney, B. G., Wagner, T. M. 1996. Importance of Staphylococcus hyicus ssp hyicus as a cause of arthritis in pigs up to 12 weeks of age. Austr. Vet. J. 73:9, 179–181

*Aerococcus* sp. was a common finding with 43 isolates both from joints with macroscopical changes and from macroscopically unaffected joints.

In the anaerobic cultivation, 24 of 130 samples (18%) were positive, including 26 bacterial species, four of which that were not possible to identify by MALDI-ToF. Three isolates were from macroscopically normal joints. The most common species found were *Clostridium (C.) perfringens* (*n* = 14), *Fusobacterium necrophorum* (*n* = 3), *Peptoniphilus (P.) indolicus* (*n* = 3), *Bacteroides (B.) pyogenes* (*n* = 1) and *B. fragilis* (*n* = 1). Of these, *C. perfringens* (*n* = 5), *P. indolicus*, (*n* = 1), *B. fragilis* (*n* = 1), and one non-typeable isolate, were from joints where no bacteria were identified by aerobic cultivation.

Sixty-four isolates of *S. hyicus* were tested for antimicrobial susceptibility (breakpoints are given below within brackets). Most (*n* = 46; 72%) of these were resistant to penicillin as indicated by beta-lactamase production. No isolate was resistant to oxacillin, thus there were no indication of methicillin resistance (Table 5).

Of the susceptibility-tested isolates of *S. dysgalactiae* ssp. *equisimilis* (*n* = 44), all were susceptible to penicillin with MICs ≤ 0.03 (MIC ≤0.5) but all were resistant to tetracycline (MIC > 1; Table 6).

**Table 5:**
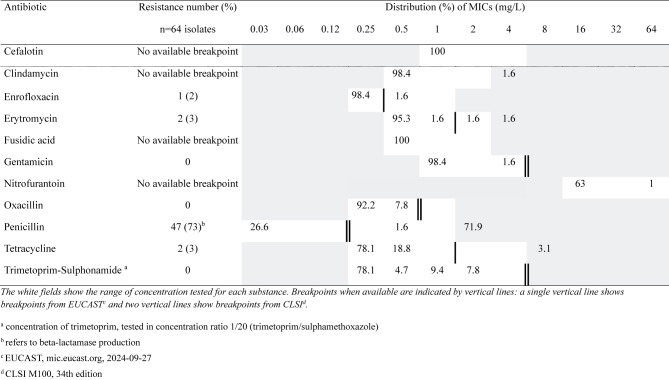
Distribution of MICs and resistance (%) in *Staphylococcus hyicus*

**Table 6:**
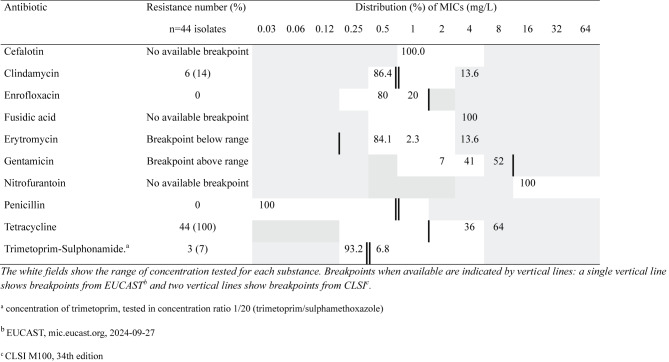
Distribution of MICs and resistance (%) in *Streptococcus dysgalactiae* ssp. *equisimilis*

One of five tested *S. suis* isolates was resistant to penicillin (MIC > 0.5), four were resistant to tetracycline (MIC > 1) and two were resistant to trimethoprim-sulphonamide (MIC > 0.25). Five out of nine isolates of *S. aureus* were classified as resistant to penicillin by beta-lactamase production. These five isolates were all susceptible to cefoxitin (MIC ≤4) and thus not methicillin resistant. The beta-haemolytic streptococci were susceptible to penicillin with MICs ≤ 0.03 (MIC ≤0.5) and two isolates were resistant to erythromycin (MIC > 0.5). Four isolates of *E. coli* were tested for antibiotic susceptibility of which two isolates were resistant to trimethoprim-sulphonamide (MIC > 2), and two to tetracycline (MIC > 8). All *Actinobacillus* sp. isolates were susceptible to penicillin, ampicillin (MIC > 1), (MIC > 4) and enrofloxacin (MIC > 0.5).

## Discussion

In this study, it should be acknowledged that the findings apply to a limited number of animals and may thus not be extrapolated to a larger population. Further, a selection bias was probably present, since the farmers most likely culled those animals that were deemed to be beyond remedy [9]. However, some issues can be pointed out:

In the present study, arthritis was the main cause of lameness in all age-categories investigated. Many studies have been performed on lame sows; however, few studies have included *post mortem* examinations [7, 9, 11]. The findings were consistent with what has previously been described, i.e. the main cause for culling of sows being related to the locomotor system, and arthritis being the main finding [9, 12, 13]. Systematic bacteriological cultivation has rarely been performed in studies on lame sows, but a few studies report findings of *T. (A.) pyogenes* [12, 13]. In the present study, most sows had been treated with antibiotics and cultivation was reckoned to be of little value. In summary, 25 sows and 32 finisher pigs had been treated with antibiotics, and the *post mortem* findings indicated an infectious aetiology in 12 and 24, respectively, of these. Thus, 13 sows (52%) and eight finisher pigs (25%) treated with antibiotics suffered from lameness inflicted by non-infectious causes, underscoring the difficulties to obtain a proper clinical diagnosis, especially in those parts of the extremities that are covered by large muscle groups. In 24 sows, penicillin was the drug of choice, which would be in accordance with current knowledge on infectious causes and antibiotic susceptibility pattern. It would have been desirable to include more non-treated sows. However, sows that might recover after treatment will not be culled and hence, this was considered as the best possible option.

In six sows (21%), the duration of lameness was reportedly less than one week. However, at necropsy, 27 of 29 sows (93%) displayed pathology including chronic lesions, 14 of which were assessed to have an infectious aetiology (Table 1). In the finisher pigs, most pigs had reportedly been lame for one week, but 32 (80%) of the 40 pigs included had chronic lesions, 31 of which were suffering from chronic arthritis, osteomyelitis and abscesses (Table 2). Thus, all sows and finisher displaying an infectious aetiology at necropsy had chronic elements included in the lesions. In the suckling pigs, 101 had reportedly been lame for one day or less; however, 78 of these (77%) suffered from chronic arthritis and in 125 piglets with arthritis being the primary diagnosis, 104 (83%) had lesions with chronic elements included. The chronicity of these lesions constitutes an animal welfare problem [4]. It is possible that the initial clinical signs of even severe joint lesions are difficult to detect. However, lesions in bone and joints are known to cause pain and thus, in accordance with the Swedish legislation on conditional medical treatment, caretakers should be educated and encouraged to spend more time observing the pigs in order to detect lameness as early as possible to facilitate early treatment and subsequent cure, hence avoiding the risk to violate animal welfare. In this respect, a confiding communication between the caretaker and the veterinarian is important. Furthermore, the effect of antibiotic treatment in chronic cases could be questioned and may in the long-term increase the risk for development of antibiotic resistance [24]. Thus, culling would in most cases have been a valid alternative.

The findings at necropsy in 7% (*n* = 2) of the sows, 11% (*n* = 5) of the finisher pigs, and 3% (*n* = 4) of the suckling pigs, did not confirm the clinical diagnosis of “lameness”. Other researchers have also found disagreements between the farmers’ perception and the findings at necropsy [9], in particular difficulties to distinguish between arthritis and fractures [11]. Thus, seven of ten sows with clinically suspected fractures at euthanasia had previously been treated with antibiotics, and three cases with fractures were not detected *ante mortem*. However, these cases should never have been treated with antibiotics but instead have been promptly euthanized.

In finisher pigs, most pathoanatomical studies have been performed on clinically healthy pigs at the abattoir [15–17, 25]. Thus, the accuracy of any treatments would be difficult to estimate. In the present study, the most common (*n* = 9) misdiagnosis was chronic arthritis, clinically suspected as fracture at the time of euthanasia. Of 11 pigs suspected to suffer from fractures, two pigs were euthanized on the day of detection, whereas nine pigs had previously been treated for arthritis by the stockmen. In accordance with previous studies, describing arthritis in 39.5%, and fractures in 7.0% of the cases [26], arthritis was the main finding in 77.5% of the present cases. In the six cases where samples were taken from affected joints for bacteriological culture, the results indicated that penicillin would be the drug of choice, although no investigations on antibacterial susceptibility were performed. Similar to the sows, however, the results of the pathological examinations indicated that treatment in most cases were initiated too late to ensure recovery. In contrast to other studies that found osteochondrosis in 88.4% of the cases, being the main diagnosis in 26% of the pigs [26], osteochondrosis was the main finding in only two of the 40 pigs examined. However, since only the most affected leg was submitted for necropsy, the overall prevalence of osteochondrosis could not be determined.

There are very few studies on the aetiology of lameness in suckling pigs. One study showed that 94.3% of 175 lame pigs suffered from arthritis [18]. In accordance with this, the present study confirmed arthritis as presumed cause of lameness in 96.9% of the cases. It is not known, if the lack of studies on suckling pigs is due to different perceptions of the problem, or if a large variation in prevalence exists between different countries, perhaps due to variations in predisposing factors.

In accordance with other studies [1, 18], infections with *S. hyicus* and *S. dysgalactiae* ssp. *equisimilis* were the most common causes of arthritis in suckling pigs, and penicillin is usually regarded as the drug of choice for treatment [22, 24]. *S. dysgalactiae* ssp. *equisimilis* were in all cases susceptible to penicillin, however, the majority of the *S. hyicus* isolates were resistant. Thus, treatment failure could be caused by treatment of irresponsive, chronic cases of arthritis, but also by the presence of resistant strains of *S. hyicus.* As the majority of the *S. hyicus* isolates were resistant to penicillin, resistance should be considered as a cause of treatment failure. However, in most cases, penicillin would be a valid first-line choice of treatment. Since antimicrobial exposure is the most important factor behind the development of antibiotic resistance, prompt, targeted treatments are imperative, and treatment of chronic, non-responsive cases should be avoided. In cases of treatment failure, the occurrence of mistreatments should be investigated by e.g. field necropsies, and sampling for bacteriology including susceptibility testing should be performed. Surprisingly many isolates of *Aerococcus* sp. were identified, although most isolates were found in a mixed bacterial flora. *Aerococcus* sp. has previously been isolated from clinical samples in pigs with lameness, however, the clinical relevance is unclear [27, 28]. Similar to the few other studies available [29], the presence of bacteria likely constituting a contaminating flora was a severe problem in the analyses in the current study. The ethanol disinfection and sterilisation by burning before incision reduced the problems but did not eliminate them. Possibly, freezing of the carcasses might have enhanced the microbial penetration of the skin, and bacterial overgrowth could have occurred during the time lapse from euthanasia to necropsy, and during thawing. Sampling by synovial-fluid aspirates in sedated animals or immediately after euthanasia might be a feasible way to reduce the level of contamination [15].

Degenerative joint disease/osteoarthritis (OA) is reportedly a common incidental finding [9, 13, 15]. Osteochondrosis with subsequent dissecans lesions of the joints develop in the growing pig [30], and OA may be seen as a secondary lesion [13, 31]. This seems to be in accordance with the findings in the present study, where OA was absent in suckling pigs and osteochondrosis dissecans solely were detected in two finisher pigs, whereas OA were found in three sows. Clinical lameness due to osteochondrosis will mainly occur in cases of osteochondrosis dissecans [8, 32, 32]. Other studies report a much higher prevalence (50–92.5%) of OA in sows [12, 13]; however, the results are not comparable, since the present investigation only included *post mortem* examination of the most severely affected leg.

To minimise the risk of lameness and the subsequent use of antibiotics, the identification of and measures undertaken to reduce predisposing factors, are of major importance. Several factors are described, in particular, factors related to the floor such as poor hygiene, worn surfaces, flooring, the width of slats and slots, and sharp-edged slats [4, 33–40], whereas the use of straw and deep-straw litter bedding seems protective [33, 41]. Factors related to genetics [5, 42, 43] may be important, and factors related to mixing and ranking-related fighting, the number of sows per pen, the space per sow, static or dynamic systems for grouping may be predisposing [35, 40, 44, 45]. Similar to other studies, most sows were less than two years of age and 40% of the cases were detected in the first few weeks post weaning [9, 44]. This may indicate that fighting and mounting behaviour could in part be responsible for the lesions seen in the loose-housed sows in the present study. Further, the claw lesions and skin abrasions noted might be caused by poor flooring. It is noteworthy that skin abrasions close to affected joints were commonly found in suckling pigs (73%), however, deep skin ulcers penetrating the dermis were only seen in 24% of the pigs, thus obscuring the interpretation. Thus, the flooring seems to be important [1] but was not targeted in the present study and warrants further investigations. Rupture of the umbilical cord could serve as portal of entrance for an infection that subsequently may be spread systemically [1, 19]. Umbilical abscesses were found in half of the suckling pigs, and of 12 pigs with abscesses > 6 mm diameter, all were suffering from polyarthritis. Similar to the skin wounds, the interpretation of this finding is challenging. In addition, studies on the presence of relapsing infections would be of value.

## Conclusion

In the majority of the cases, the findings at necropsy confirmed the clinical diagnosis. It should however be acknowledged that the cause of lameness may be hard to establish. In particular, chronic arthritis and fractures may be difficult to differentiate in large animals. The results from the present study also indicate the occurrence of inappropriate antibiotic use in the treatment of lame pigs, since 21 of 57 sows and finisher pigs (37%) treated with antibiotics suffered from lameness inflicted by non-infectious causes. Further, summarising the findings, all sows and finisher pigs (14 sows and 31 finisher pigs) and 83% (*n* = 104) of the suckling pigs with lesions being assessed to have an infectious aetiology at the *post mortem* examinations, were interpreted as being chronic. Pigs suffering from fractures should be promptly euthanized and treatment of these animals, as well as delayed treatments of pigs suffering from arthritis, constitute an animal welfare issue. In piglets, *S. dysgalactiae* ssp. *equisimilis* or *S. hyicu*s were isolated in 64% of the samples. A majority of these isolates were susceptible to penicillin, still rendering this a valid drug for treatment in acute cases of arthritis.

## Data Availability

No datasets were generated or analysed during the current study.
